# Turbulent Aggregation and Deposition Mechanism of Respirable Dust Pollutants under Wet Dedusting using a Two-Fluid Model with the Population Balance Method

**DOI:** 10.3390/ijerph16183359

**Published:** 2019-09-11

**Authors:** Pei Wang, Shuai Shen, Ling Zhou, Deyou Liu

**Affiliations:** 1 Department of Renewable Energy, Hohai University, Nanjing 210098, China; shenshuai23@hhu.edu.cn; 2 College of Water Conservancy and Hydropower Engineering, Hohai University, Nanjing 210098, China; zlhhu@hhu.edu.cn (L.Z.); liudyhhuc@163.com (D.L.)

**Keywords:** turbulence aggregation, deposition, wet dedusting, respirable dust, population balance model, multiphase flow

## Abstract

In this paper, a mathematical model based on the two-fluid (Euler–Euler) frame model for wet dedusting process is proposed. The model considers in detail the aggregation of particles and droplets caused by turbulence and Brownian diffusion as well as the gravitational deposition process. The population balance model (PBM) is used to describe the spatiotemporal evolution of particle size distribution (PSD) for the dust particle and the water droplet. The wet dedusting process under different conditions is simulated and compared with the detailed experimental data. The results show that the experimental data and simulation results are within the allowable range of error (about 32.3–61.2% in dedusting efficiency for respirable dust by experimental data and about 47.3–57.9% in it by simulation results). This model can be used to predict the effect of PSD of the dust particle, spray flow, and ventilation rate on dedusting efficiency of wet dedusting. The parameter analysis shows that dedusting efficiency decreases as particle size decreases. In order to ensure high capture efficiency of respirable dust, the diameter of droplets should be controlled to between 15 μm and 70 μm. The ratio of droplet volume flow to dust volume flow increases from 2.0 to 12.0, while dedusting efficiency only increases from 39.2% to 54.7%, so it is clear that for spray quantity to dedusting efficiency, larger is not necessarily better. Besides this, the speeds of both spray droplets and ventilation also have great influence on dedusting efficiency, and the related formulas are given.

## 1. Introduction

At present, many industries that need to carry out large-scale construction and transformation of underground space are developing rapidly. It is well established that tunnels, hydroelectric power stations, and coal mines produce large amounts of dust when they are built or mined, and inhalation exposure to airborne particles, especially respirable dust (aerodynamic diameter below 7.07 microns), can have adverse effects on health. The main technology worldwide for the prevention and control of respirable dust is external water-spray in working face; however, if high levels of instant dust groups are not arrested and settled in the first place, dust can easily spread [1]. At present, wet dedusting technology itself is relatively mature, and its engineering application research results are scattered in many engineering fields, such as mining, construction, transportation, municipal engineering and so on [2,3,4,5]. However, there is still a lack of a complete set of theoretical system. In particular, under the condition of ventilation, the relevant mechanism and engineering application of dust–droplet–air multiphase flow system containing particles involved in the process of adjusting dust and other environmental parameters by spray dedusting technology have not been reported. In light of this, solutions are urgently needed for the problems of construction and working environment in enclosed spaces. Consequently, we need to model and analyze the water-spray method of dedusting in both small and large contained spaces.

Liang’s [6] experimental research focused on the different cooperation modes of eddy–current dust control systems and wet swirling dust removal systems and obtained the best parameters affecting dust removal efficiency, including the distance between dust removal device and working face, gap width of air duct outlet, and ventilation volume. Nie [7] determined atomized particle size at the heading face for two common pressure nozzles affected by 0.4 m/s airflow and observed that increased pressure, reduced axial distance from the nozzle, and reduced distance from the axial center line of the nozzle will cause the atomized droplet size to decrease. Wang [8] tested the dedusting effects of spiral pressure nozzles under different spray pressures, nozzle diameters, and roadway wind speeds. He found that dedusting efficiency increased with an increase in nozzle pressure and the same water consumption and that dedusting efficiency decreased with an increase in nozzle diameter. Nie [9] developed an experimental platform that measures performance with regard to spray atomization and dust removal, analyzing the performances of 22 kinds of nozzles.

Rigorous theoretical studies of the capture process of dust particles by water droplets have been conducted on a mesoscopic scale. Dubrovsky et al. [10] carried out extensive experiments on droplet–particle collision using particles and droplets of different sizes and physical properties, and they identified four different interaction regimes: capture, shooting through, bubble formation, and target destruction. Mitra et al. [11] studied the collision process using high speed video imaging involving glass ballotini particles and a supported stationary water droplet at different particle-impact velocities (Weber number range: 0.2–13.5). However, for ventilation design or dust control in engineering applications like complex terrain or underground caverns, robust mathematical analysis has not been reported. Some studies focus on a comprehensive analysis of the water-spray mechanism of dust suppression from a mesoscopic scale [12,13,14]. It is believed that the reason water mist can catch dust flying in the air is the comprehensive mechanisms such as inertial collision, interception, gravity, diffusion, eddy current aggregation, etc. [15]. However, no one has yet come up with detailed results for the more systematic coupling theory of dust and water mist.

In terms of numerical simulation and application generally, there are two methods, namely the Euler–Lagrange method and the Euler–Euler method. As for the Euler–Lagrange method, Zhang [16] used a discrete-phase model to simulate the atomization effect of spray devices under different parameters. Wang [17] successfully established three-dimensional (3D) steady wind and spray flow models and investigated the influence of airflow from forced ventilation on the water spray flow field. Zhou [18] employed computational fluid dynamics (CFD) to conduct a numerical simulation on the airflow–dust field and airflow–droplet field in the fully-mechanized mining face in order to ascertain the migration and distribution rules of dust particles and droplets under air flow. Yu [19] established an airflow–droplet–dust three-phase coupling model based on the CFD-DEM (Discrete Element Method) model and investigated the dust suppression rules under different spraying schemes.

As for the Euler–Euler method, Hong [20] described a 3D unsteady two-phase turbulence model considering the effect of gas–solid interaction force and analyzed airflow structure and dust migration. In order to solve PBM [21] so that it can be used in numerical simulation, Hounslow [22], Litster [23], and Ramkrishna [24] developed the discrete method (also known as the classes or sectional method), which is based on representing the continuous PSD in terms of a set of discrete size classes or bins. There have been several studies of the theoretical evaluation of the deposition rate of aerosols due to Brownian and turbulent diffusion accompanying gravitational sedimentation in a closed chamber (Corner and Pendlebury; Crump and Seinfeld; Park et al.) [25,26,27]. Jung and Lee [28] analyzed the collection of small particles by a system consisting of multiple fluid spheres such as water droplets or gas bubbles. Using the resolved flow field, they obtained analytic solutions for the particle collection efficiencies due to diffusion and interception. Using a novel PBM framework, Barrasso [29] developed a multi-dimensional, multi-component model for a continuous granulation process, describing time evolutions of distributions with respect to granule size, liquid distribution, and granule composition. Akbari [30] studied the segregation of a wide range of PSDs in an industrial gas phase polymerization reactor by means of a CFD–PBM coupled model in which the direct quadrature method of moments (DQMOM) was implemented to solve the PBM.

A survey of existing research into spray dedusting reveals that the basic theory and the experimental research are relatively detailed. The discrete particle method (DPM) and the discrete element method (DEM) based on the Euler–Lagrange frame are adopted by researchers in this field to simulate spray dedusting; the DPM and the DEM can study particle trajectories and collisions with each other through tracing [31], however, they require a lot of computing resources and are difficult to apply to large-scale computing in engineering field. There is widespread theoretical research being conducted on the PBM method based on the two-fluid (Euler–Euler) frame. The PBM can characterize the variation of number density of target particles with internal properties, spatial location, and external time in the form of probability density function. It has been widely used in predicting the size distributions of discrete particles including aggregation, fragmentation, and nucleation behavior, but it has not yet been applied to wet dedusting.

This study proposes a Euler–Euler model coupled with the PBM to describe the process of spray dedusting. The model considers in detail the aggregation and gravitational deposition processes between particles and droplets caused by turbulence and Brownian diffusion. The PBM is used to describe the spatiotemporal evolution of particle-size distribution and droplet-size distribution. The numerical simulation results were compared with the experimental data in literature, and the model exhibits a certain accuracy and potential in the accurate prediction of dedusting efficiency.

## 2. The Numerical Model of Spray Dedusting

### 2.1. Two-Fluid (Euler–Euler) Model

The particle size of dust and droplets is very small, and they are sparsely distributed in space; however, the interaction between particles should be taken into account. Therefore, the TF model (primary phase, the air and secondary phase, the particle) is used to calculate the velocity field. In this model, the air phase can be considered as a continuous phase in the calculation area, while the droplets and dust particles should be considered as a discretized phase, and the interaction between the phases can be accounted for using the drag force model.

The conservation equations of mass and momentum can be written as

(1) $$\frac{\partial }{\partial t}\left(\varphi \rho \right)+\nabla \cdot \left(\varphi \rho v\right)=0$$

(2) $$\frac{\partial }{\partial t}\left(\varphi \rho v\right)+\nabla \cdot \left(\varphi \rho vv\right)=-\varphi \nabla p+\nabla \cdot \bar{\tau }+\varphi \rho g+\sum_{p=1}^{2}R_{p}+\left(F+F_{vm}\right)$$

where *φ* is the volume fraction of each phase, *ρ* is phase density, *v* is phase velocity, ∇*p* is pressure gradient, $\bar{\tau }$ is stress tensor, *φρ**g*** is gravity term, ***g*** is gravity acceleration, $\sum_{p=1}^{2}R_{p}$ is interphase drag term, ***F*** is additional physical force, and ***F****_vm_* is virtual mass force.

The components of multiphase flow in this paper are air, dust particles and water mist, and turbulence plays an important role in the aggregation of particles. Compared with the standard *k*-*ε* model, the realizable *k*-*ε* model introduces the correlation terms of rotation and curvature to avoid negative normal stress, so the realizable *k*-*ε* turbulence model was applied [32]. The transport equations of turbulence kinetic energy *k* and dissipation rate *ε* are as follows:

(3) $$\frac{\partial }{\partial t}\left(\rho k\right)+\frac{\partial }{\partial x_{i}}\left(\rho ku_{i}\right)=\frac{\partial }{\partial x_{j}}\left[\left(\mu +\frac{\mu_{t}}{\sigma_{k}}\right)\frac{\partial k}{\partial x_{j}}\right]+G_{k}-\rho \varepsilon$$

(4) $$\frac{\partial }{\partial t}\left(\rho \varepsilon \right)+\frac{\partial }{\partial x_{i}}\left(\rho \varepsilon u_{i}\right)=\frac{\partial }{\partial x_{j}}\left[\left(\mu +\frac{\mu_{t}}{\varepsilon_{k}}\right)\frac{\partial \varepsilon }{\partial x_{j}}\right]-\rho C_{2}\frac{\varepsilon^{2}}{k+\sqrt{\varepsilon v}}$$

where *ρ* is fluid density, *x_i_* and *x_j_* are the components of coordinates, *σ_k_*, *σ_ε_* are turbulent Prandt numbers of turbulent kinetic energy *k* and dissipation rate *ε*, *G_k_* is the turbulent kinetic energy generated by the average velocity gradient, *μ* is the coefficient of molecular viscosity, *μ_t_* is turbulence viscosity coefficient, *v* is the coefficient of kinetic viscosity, *C*_2_ is an empirical coefficient.

### 2.2. Population Balance Model

PBE model is used to calculate the aggregation effect of particles in this paper, and the phenomena of nucleation, growth, and breakup of particles are excluded. Based on the hypothesis of particle sparsity and molecular chaos, the zero-dimensional equilibrium equation of PSD function in the Eulerian coordinate system with only particle aggregation considered can be written as [33]

(5) $$\frac{\partial n\left(\nu ,t\right)}{\partial t}=\frac{1}{2}\int_{\nu_{\min}}^{\nu }\beta \left(\nu -u,u,t\right)n\left(\nu -u,t\right)n\left(u,t\right)du-n\left(v,t\right)\int_{\nu_{\min}}^{\nu_{\max}}\beta \left(\nu ,u,t\right)n\left(u,t\right)du$$

where *n*(*v*, *t*) denotes the number density function of particles with volume *v* at time *t*, using the unit 1/m^3^; *β*(*u*, *v-u*) denotes the aggregation nucleus of particles with volume *u* and *v-u*, using the unit m^3^/s; the first term on the right side of the equation denotes the number of new particles with volume *v* generated by aggregation, and ½ denotes that two particles participate simultaneously in a single aggregation event; the second term denotes the number of particles whose volume vanishes as *v* as a result of aggregation into larger particles.

Generally, the PBE can be solved by two types of methods: the discrete method and the method of moments. Because this study has certain requirements regarding PSDs and their respective volume fraction ratios, the more advantageous inhomogeneous discrete method is adopted. The advantages of this method are its robust numerics and the fact that it gives the PSD directly. The disadvantages are that the bins must be defined a priori and that a large number of classes may be required. One of the limitations of the existing homogeneous discrete method is that all bins are assigned to the same secondary phase and are therefore advected with the same phase momentum. This is unsuitable for modeling cases in which large and small bin sizes are likely to segregate due to different momentum fields. The inhomogeneous discrete method overcomes this limitation by allowing groups of bins to be advected by different phase velocities. The solution methods for the inhomogeneous discrete method are based on the discrete method and therefore share many of the same fundamentals. In the discrete method, the PBE is written in terms of volume fraction of particle size:

(6) $$\frac{\partial }{\partial t}\left(\rho_{S}\alpha_{i}\right)+\nabla \cdot \left(\rho_{S}u_{i}\alpha_{i}\right)=\rho_{S}V_{i}\left(B_{ag,i}-D_{ag,i}\right)$$

where *ρ_s_* is the density of the secondary phase and *α_i_* is the volume fraction of particle size *i*, defined as

(7) $$\alpha_{i}=N_{i}V_{i}\;i=0,1,\cdots ,N-1$$

where

(8) $$N_{i}\left(t\right)=\int_{V_{i}}^{V_{i+1}}n\left(V,t\right)\;dV$$

and *V_i_* is the volume of the particle size *i*. Then a fraction of *α_i_* called *f_i_* is introduced as the solution variable. This fraction is defined as

(9) $$f_{i}=\frac{\alpha_{i}}{\alpha }$$

where α is the total volume fraction of the secondary phase. The particle birth and death rates by the aggregation are defined as follows:

(10) $$B_{ag,i}=\sum_{k=1}^{N}\sum_{j=1}^{N}a_{kj}N_{k}N_{j}x_{kj}\xi_{kj}$$

(11) $$D_{ag,i}=\sum_{j=1}^{N}a_{ij}N_{i}N_{j}$$

where

(12) $$a_{ij}=a\left(V_{i},V_{j}\right)$$

and

(13) $$\xi_{kj}=\left\{ \begin{array}{cc} 1 & \mathrm{for}\;V_{i}<V_{ag}<V_{i+1},\mathrm{where}\;i\leq N-1\\ 0 & \mathrm{otherwise} \end{array}\right.$$

*V_ag_* is the particle volume resulting from the aggregation of particles *k* and *j*, and is defined as

(14) $$V_{ag}=\left[x_{kj}V_{i}+\left(1-x_{kj}\right)V_{i+1}\right]$$

where

(15) $$x_{kj}=\frac{V_{ag}-V_{i+1}}{V_{i}-V_{i+1}}$$

If *V_ag_* is greater than or equal to the largest particle size *V_N_*, then the contribution to class *N* − 1 is

(16) $$x_{kj}=\frac{V_{ag}}{V_{N}}$$

### 2.3. Aggregation Kernel Model

The aggregation mechanisms include free molecular aggregation, coulomb aggregation, turbulence aggregation, bubble aggregation, and droplet aggregation, which can be seen in Table 1. In practical engineering applications, the droplet size produced by various atomizers is larger than 10 μm [34], so there is no aggregation caused by Brownian motion between droplet particles. Although the aggregation between dust particles occurs when the local humidity increases to a certain value, the Brownian aggregation between dust particles less than 1μm can still be neglected compared with the capture of dust by droplets. Therefore, the free molecular aggregation model is not discussed in this paper. Considering that the respirable dust and liquid droplets involved in this study are not charged, the coulomb aggregation model is not applicable.

The bubble aggregation model and the droplet aggregation model assume that the average turbulent distance of particles is larger than the particle spacing, but the particle spacing is larger when the particle phase is relatively sparse. When the particle spacing is larger than the turbulent distance, the calculated value of particle collision frequency is larger than the actual value. The turbulent aggregation model divides the aggregation mechanism into cohesive aggregation and inertial aggregation according to the relationship between particle diameter and minimum vorticity size (Kolmogorov microscale) and considers them separately so as to obtain more accurate particle aggregation efficiency and size distributions [42].

For the reasons discussed above, only the turbulent aggregation model was selected for use in this study. In the turbulent aggregation model, aggregation between particles is dominated by viscous and inertial mechanism. Under the viscous mechanism, particle collisions are influenced by the local shear within the eddy. The aggregation rate of particles is expressed as in Reference [43]

(17) $$a\left(d_{i},d_{j}\right)=\varsigma_{T}\sqrt{\frac{8\pi }{15}}\gamma \frac{{\left(d_{i}+d_{j}\right)}^{3}}{8}$$

where *ς_T_* is a pre-factor that takes the capture efficiency coefficient of turbulent collision into account; *γ* denotes the shear rate, and it can be written as

(18) $$\gamma =\frac{\varepsilon^{0.5}}{v}$$

Under the inertial mechanism, particles are bigger than the smallest eddy, therefore they are dragged by velocity fluctuations in the flow field. In this case, the particle aggregation rate can be expressed as [44]

(19) $$a\left(d_{i},d_{j}\right)=\varsigma_{T}2^{3/2}\sqrt{\pi }\frac{{\left(d_{i}+d_{j}\right)}^{2}}{4}\sqrt{\left(U_{i}^{2}+U_{j}^{2}\right)}$$

where *U_i_*^2^ denotes the mean square velocity for particles *i*. The expression of the empirical capture efficiency coefficient of turbulent collision can be written as [45]

(20) $$\varsigma_{T}=0.732{\left(\frac{5}{N_{T}}\right)}^{0.242}\left(N_{T}\geq 5\right)$$

where *N*_T_ is the ratio between the viscous force and the Van der Waals force:

(21) $$N_{T}=\frac{6\pi \mu {\left(d_{i}+d_{j}\right)}^{3}\dot{\lambda }}{8H}$$

where *H* is the Hamaker constant, a function of the particle material; $\dot{\lambda }$ denotes the deformation rate:

(22) $$\dot{\lambda }={\left(\frac{4\varepsilon }{15\pi v}\right)}^{0.5}$$

In particular, the PBM is impossible to distinguish droplets from dust particles. Therefore, the statistical model must be modified according to the dynamic characteristics of different physical properties and particle sizes in order to obtain an accurate description of particle behavior. On this issue, because the diameter of water mist particles is much larger than that of dust particles, and there is no intersection between the two particle size distributions from beginning to end, we distinguish the two particles in the same phase through the difference of bins, in order to achieve the statistical analysis of the particle size distribution of two different kinds of particles. The density, viscosity, and surface tension coefficients of dust and droplets are not taken into account directly. Judgment statements are added to define the aggregation after collision of dust particles and water droplets, which avoids the phenomenon contrary to reality that particle–particle coalesce and droplet–droplet coalesce at the same time.

### 2.4. Deposition Kernel Model

In most of the previous work, the deposition process of dust particles are neglected. Actually, in the absence of electrical and thermal forces, gravitational deposition is the most important effect mechanisms in large spatial and temporal scale. The deposition coefficient for a chamber of arbitrary shape is represented by the following equation, which accounts for turbulent and Brownian diffusion and gravitational deposition [46]:

(23) $$\beta \left(r\right)=\frac{nS\sin(\pi /n)\sqrt[{n}]{k_{e}L_{2}^{2-n}D^{n-1}}}{\pi V}+\frac{u_{t}}{H}$$

where *r* is the particle radius, *D* is the Brownian diffusion coefficient, *k_e_* is the coefficient of the turbulent diffusivity (which can be evaluated from the turbulent energy dissipation rate), *L*_2_ is the turbulent boundary layer thickness [47], *n* is an experimentally-fitted parameter, *S* is the total surface area of the chamber, *V* is volume of the chamber, and *H* is the height of the chamber. *D* and *u_t_* depend on the particle size as follows:

(24) $$D=\frac{k_{B}TC}{6\pi \mu r}$$

(25) $$u_{t}=\frac{2r^{r}\rho gC}{9\mu }$$

where *k*_B_ is the Boltzmann constant, *T* is the absolute temperature, *μ* is the gas viscosity, *ρ* is the particle density, *g* is the gravity constant, and *C* is the slip correction factor, which is represented as [48]

(26) $$C=1+\frac{\lambda }{r}\left\{1.142+0.558\exp\left(-\frac{0.999r}{\lambda }\right)\right\}$$

where *λ* is the mean free-path length of the gas molecules. For a particle with a radius much smaller than *λ*, there is the following approximation:

(27) $$C\approx \frac{1.7\lambda }{r}\;\mathrm{for}\;r\ll \lambda .$$

On the other hand, for a particle with a radius much larger than *λ*, the slip correction factor can be neglected. Applying these two approximations to the first and second terms of Equation (23), respectively, Equation (23) is rewritten as follows:

(28) $$\beta \left(r\right)=\beta_{d}\left(r\right)+\beta_{g}\left(r\right)=A\cdot r^{-2(n-1)/n}+B\cdot r^{2}$$

where

(29) $$A={\left(\frac{1.7\lambda k_{B}T}{6\mu H}\right)}^{\left(n-1\right)/n}\left(\frac{nS\sin(\pi /n)\sqrt[{n}]{k_{e}L_{2}^{2-n}}}{\pi V}\right)$$

(30) $$B=\frac{2\rho g}{9\mu H}$$

The subscripts *d* and *g* refer to the diffusion term and the gravitation term, respectively. Equation (28) will be used in this study as an approximate deposition coefficient of particles in a closed chamber to add to the modeling.

Further analysis of Equation (28) shows that the particle deposition coefficient in a specific space is only a function of particle size. The coefficient multiplied by the current total number of particles is the number of particles lost per second due to deposition. Therefore, negative source terms of mass of dust particles in each bin were set to observe particle extinction due to deposition during dedusting.

The dust volume fraction source term of bin *i* (*i* = 1, 2, …, *n*) can be expressed as

(31) $$S_{V_{i}}=-\beta \left(r_{i}\right)\cdot \alpha_{i}$$

When solving the PBM, the general transport equation is calculated using the discrete bin fraction *f_i_*, so the dust volume fraction source term of each bin in the PBE can be further rewritten as

(32) $$S_{f_{i}}=-\frac{S_{V_{i}}}{\sum_{i=1}^{n}S_{V_{i}}}$$

The dust mass source term of bin *i* can be expressed as

(33) $$S_{M_{i}}=-\beta \left(r_{i}\right)\cdot \rho \cdot \alpha_{i}$$

The total mass source term of dust can be expressed as

(34) $$S_{M}=\sum_{i=1}^{n}S_{M_{i}}$$

where *α_i_* is the volume fraction of particles in bin *i*, and *ρ* is the density of dust particles.

**Figure 1:**
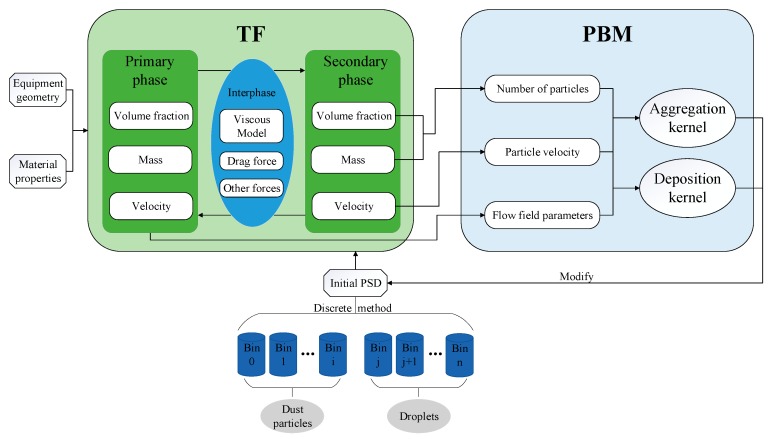
Schematic of TF-PBM model and data transfer in this study.

Figure 1 shows the structure, coupling relationship and solving process of the TF-PBM model (Euler two-fluid model and population balance model) described above.

## 3. Model Validation

### 3.1. Experimental Set

**Figure 2:**
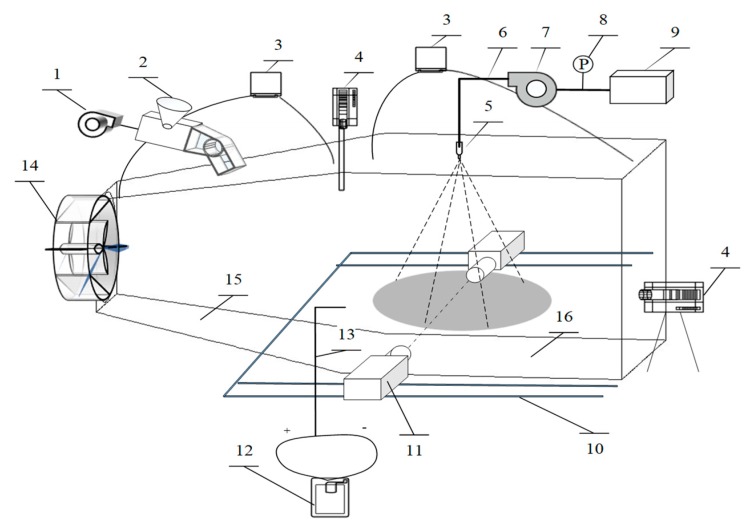
Schematic diagram of experimental model for spray dedusting. 1-air pump; 2-dust generator; 3-YJB-2500-type compensatory micro-manometer; 4-AKFC-92A-type dust meter; 5- nozzle; 6-high-pressure pipe; 7-high-pressure pump; 8-pressure gauge; 9-water tank; 10-slippery course; 11-laser particle size analyzer; 12-YYT-2000-type inclined micro-manometer; 13-Pitot tube; 14-axial flow fan; 15-airflow expanding section; 16-airflow stationary section.

The schematic diagram of the experimental model in the literature [49] is shown as Figure 2. It is primarily composed of two parts, the inlet diffuser with a shape of four-sided platform and the rectangular experimental segment with length, width, and height of 3.0 m, 3.0 m, and 2.5 m respectively. The length of the inlet diffuser is 2.75 m and the diffuser angle is 22.6°. The inlet and outlet are not closed; the other eight surfaces are sealed with plexiglass with a thickness of 1 cm. The inlet is a circle with a diameter of 1.4 m, and the outlet is a rectangle with a section of 3.0 m × 2.5 m.

**Figure 3:**
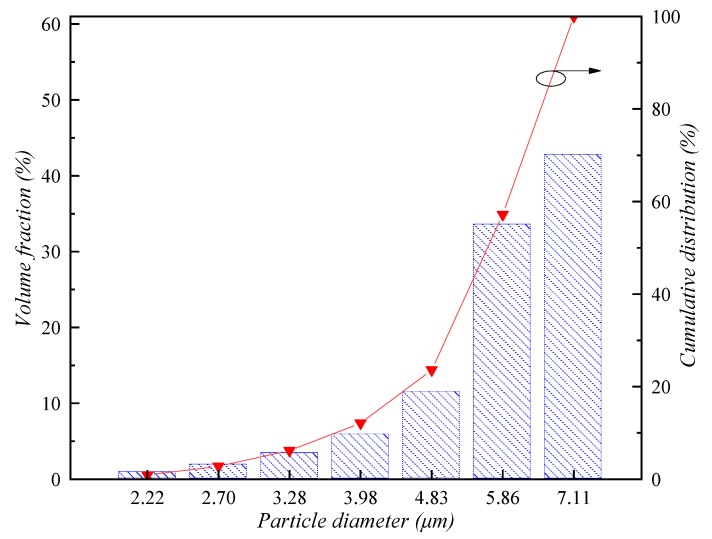
Schematic diagram of the volume fraction and its cumulative distribution of respirable dust in different particle sizes from the literature.

At this stage, we used the professional CFD software FLUENT (version 19.2, 1987–2018 ANSYS, Canonsburg, PA, USA) to simulate this experimental model. The mathematical model described above was adopted and we embedded the non-commercial code written by ourselves based on the software’s internal numerical method and code structure for calculation. All the boundary conditions were set according to experimental data in the literature. The PSD according to the experimental data were divided into seven bins (see Figure 3). The measurement data for droplet size in droplet-fields formed by different nozzles are given, including D10, D50, D90, Sauter mean diameter, and basic parameters of nozzles. We collated and selected a series of available data, which are listed in Table 2. According to these data, the particle size distribution of droplets can be divided into multiple bins in the model, and the volume fraction of particles in each bin can be set accordingly. Pressure-based solver is used in CFD calculation, SIMPLEC algorithm is used for pressure-velocity coupling term, second-order central difference scheme for pressure discretization term in control equation, second-order upwind difference scheme for momentum discretization term, and first-order scheme for turbulent kinetic energy and turbulent dissipation rate [50]. Transient simulation is carried out with a time step of 0.001 s. The convergence criterion of all scalars requires that the normalized residuals be less than 10^−6^.

### 3.2. Results and Comparison

**Figure 4:**
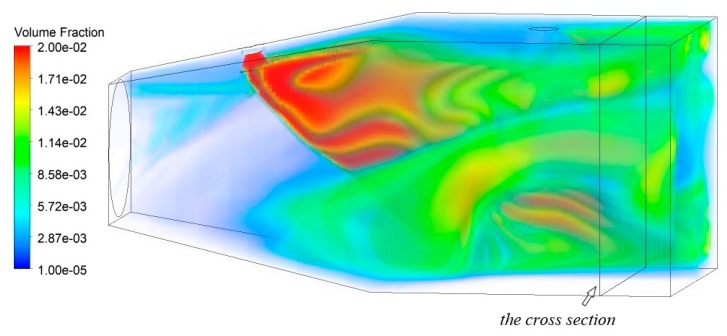
Volume rendering of dust particle volume fraction.

**Figure 5:**
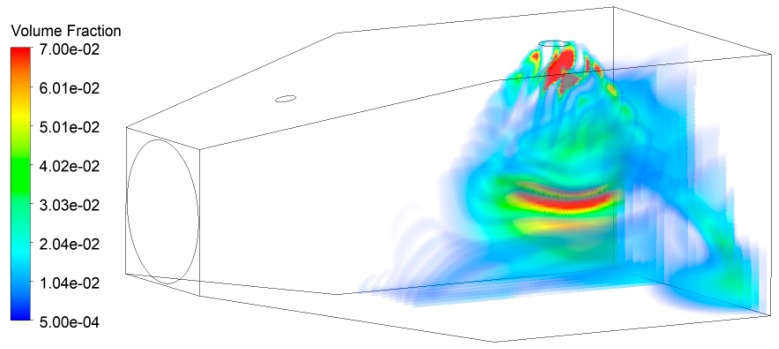
Volume rendering of water droplet volume fraction.

In case 1, the flow field and the volume fraction of the granule phase were numerically simulated by using various parameters of nozzle type 1, as shown in Figure 4 and Figure 5. Due to the axial flow fan arranged on the left side of the model, dust particles migrate to the right after entering the area and diffuse in the widening section. When dust meets droplets in the stable section of airflow, partial dust will be captured by droplets, resulting in a decrease in the volume fraction of dust particles. The fraction distribution diagrams of the rest cases of dust particles are similar to Figure 4 and Figure 5, so they will not be described here.

**Figure 6:**
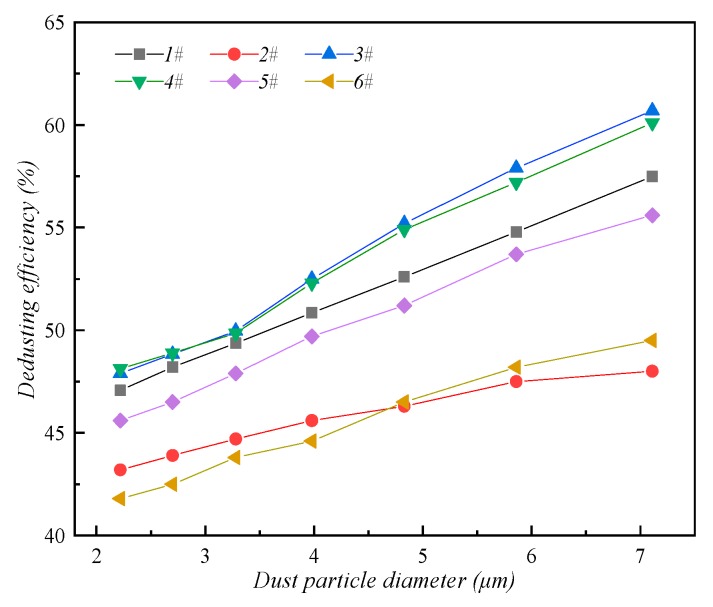
Dedusting efficiency of water-spray about different types of nozzles.

Figure 6 shows a comparison of the dedusting efficiency of water-cloud by different types of nozzles. For the particle size grouping of dust studied in this paper, the larger the particle size is, the more easily it is captured by droplets and the higher the dedusting efficiency. The reason for this phenomenon is that the collision probability and aggregation efficiency of small size dust and large size droplets are relatively low.

**Figure 7:**
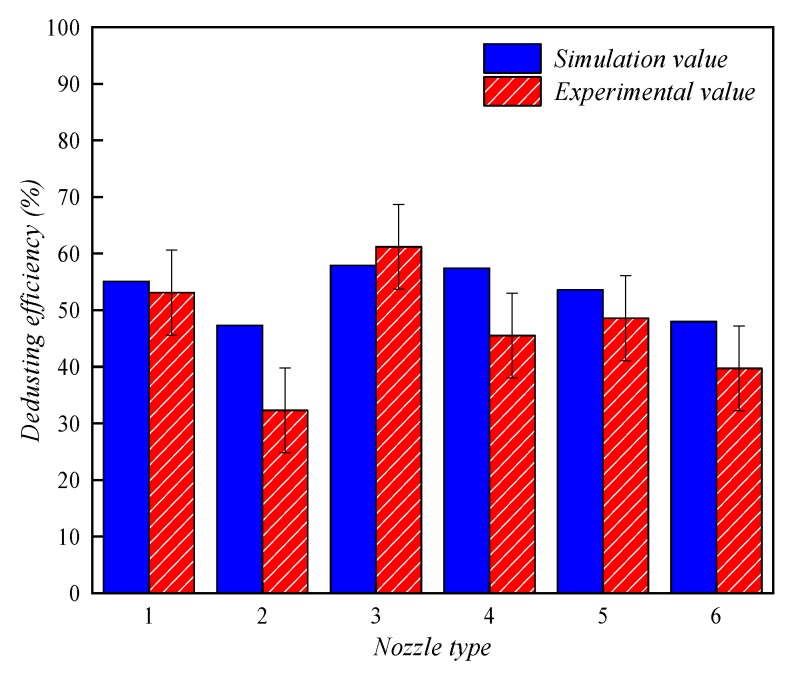
Comparison between simulation and experimental values of dedusting efficiency.

Figure 7 shows the comparison between simulation and experimental values of dedusting efficiency. Experimental data in the literature [49] show that the dedusting efficiency of six different types of nozzles for respirable dust ranges from 32.3% to 61.2%. The efficiency values simulated by the model presented in this paper are in good agreement with the experimental results within the error limits of the experiment (about 32.3–61.2% in dedusting efficiency for respirable dust by experimental data and about 47.3–57.9% in it by simulation results). However, there are still some deviations between numerical simulation and experiment, in part because the simulation does not accurately represent the specific shape of the droplet-field formed by each nozzle, which would require such measures as atomization angle, uniformity, and droplet velocity. And the small changes in nozzle shape could lead to large deviations using CFD. In addition, dust particle deposition is considered in this paper, which may also have some influence on the results. In general, the model proposed in this study has high accuracy and application value in calculating spray dedusting efficiency.

## 4. Discussion

### 4.1. Effect of Droplet Size

**Figure 8:**
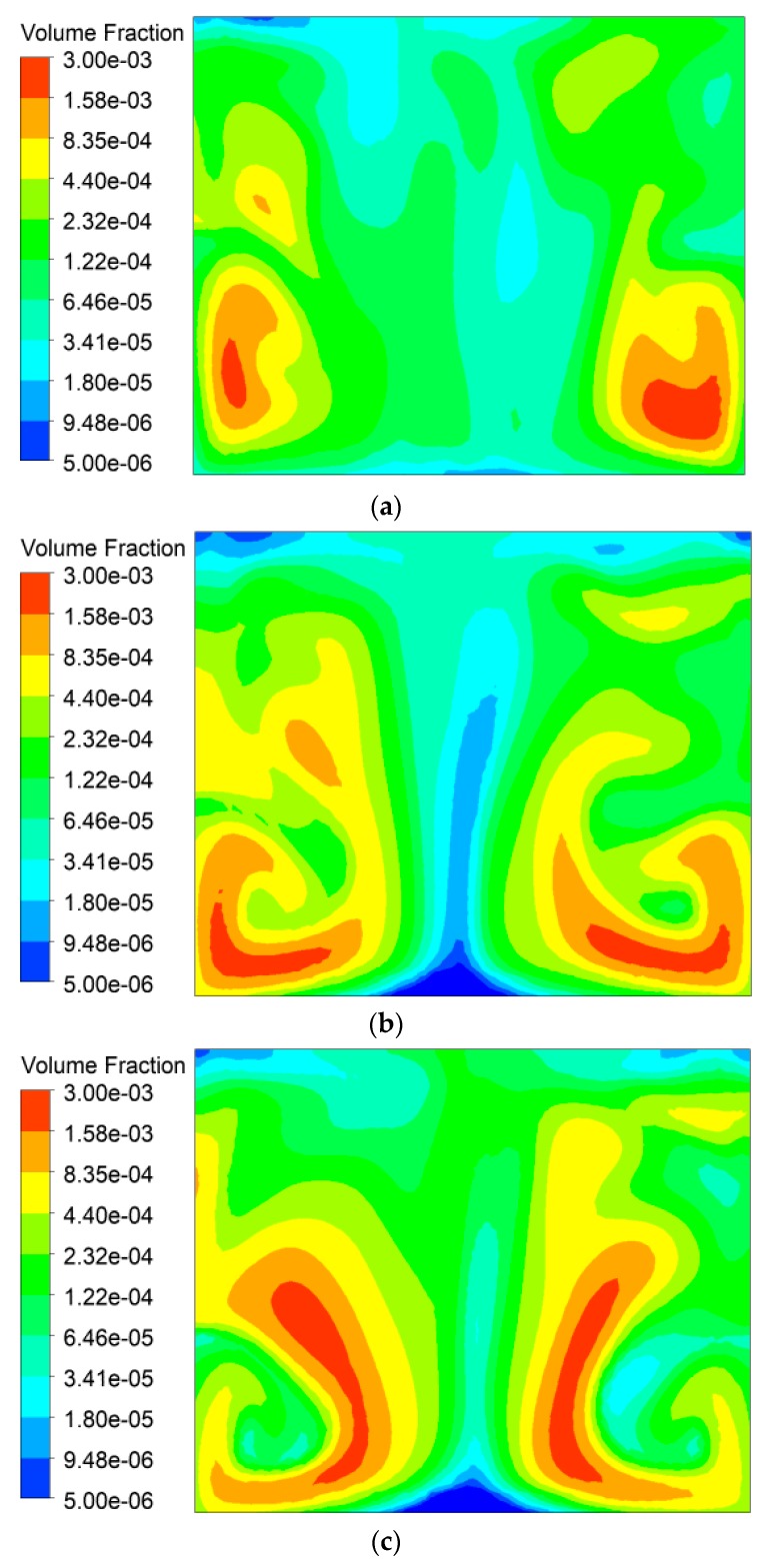
Distribution of dust particle volume fraction. (**a**) Group 1. (**b**) Group 2. (**c**) Group 3.

Droplets were divided into three groups according to the particle size: 15.3 µm and 24.3 µm in group 1, 38.6 µm and 61.3 µm in group 2, and 97.3 µm and 154 µm in group 3. The distribution of the volume fraction of dust particles with different particle sizes is shown in Figure 8. As can be seen in Figure 4, the plane is located at 0.45 m downstream of the nozzle with a coordinate of *x* = 4.7 m. The volume fraction distribution of dust is no longer uniform when the dust flows through the spray cross section in which the concentration of droplets is greatest, the dust concentration here is generally lower than that on both sides. Of the three groups, group 1 has the smallest droplet diameter; therefore, under the same nozzle mass flow rate, the number of droplets is largest and the dispersion is best, so that the spatial distribution of dust in Figure 8a is more uniform and the concentration lower than that in Figure 8b,c. Correspondingly, the dust concentration of group 3 with the largest droplet diameter is the highest, that is, the dedusting effect is the worst.

**Figure 9:**
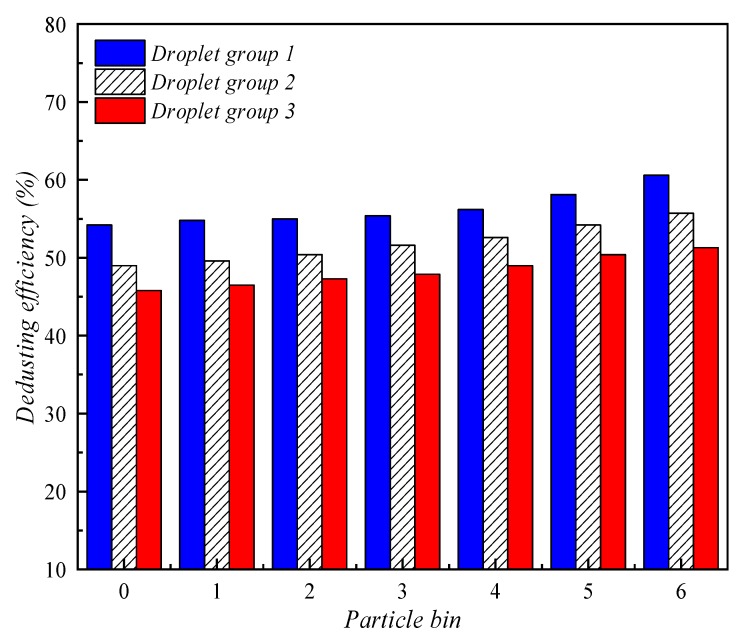
Comparison of dedusting efficiency of different droplet groups.

Figure 9 compares the dedusting efficiency of these three groups of droplets. The particle sizes represented by bins 0~6 of dust particles are 0.380 µm, 0.603 µm, 0.958 µm, 1.520 µm, 2.410 µm, 3.830 µm, and 6.080 µm, respectively. As seen in Figure 8, group 1 has the highest dedusting efficiency, group 2 has the second highest dedusting efficiency, and group 3 has the worst dedusting efficiency. In other words, within the range of water mist particle size (about 15 µm to 150 µm) produced by general spray devices, the smaller the particle size of liquid droplets, the higher the efficiency of capturing respirable dust. The reason for this phenomenon is that under the action of air resistance and gravity, water mist with smaller particle size can stay in the air longer, which can increase the probability of collision with dust particles. In addition, under the action of the same group of droplets, the dust capture efficiency also increases slightly with the increase in dust particle diameter.

### 4.2. Effect of Droplet–Particle Volume Flow Rate Ratio

The droplet–particle volume flow rate ratio *r*_f_ was adjusted with other parameters unchanged. Table 3 shows the effect of *r*_f_ on the dedusting efficiency. When *r*_f_ increased, the dedusting efficiency *η*_f_ increased significantly (from 39.2% to 54.7%). However, its increase slowed down when the *r*_f_ increased from 7 to 9. When *r*_f_ is larger than 9, the effect of increasing *r*_f_ can be neglected, and the value finally stabilizes at about 58.5%. This is because when the volume flow rate of spray increases, the number of droplets increases, resulting in a significant increase in the probability of collision between dust particles and droplets. Therefore, the aggregation efficiency of particles and droplets increases noticeably. However, considering that the number of dust particles and the size of the mixing area are certain, when the number of droplets increases to a certain extent, some droplets will become redundant. At this time, the increase of spray volume has little effect on the growth of dedusting efficiency. The fitted formula for the dedusting efficiency *η*_f_ and the droplet–particle volume flow rate ratio *r*_f_ is given in Equation (35).

### 4.3. Effect of Droplet–Particle Velocity Ratio

The droplet–particle velocity ratio *r*_v_ was adjusted with other parameters unchanged. Table 4 shows the effect of *r*_v_ on the dedusting efficiency. The dedusting efficiency *η*_v_ is only 53.1% when *r*_v_ is 1. As the *r*_v_ increases to 6, the *η*_v_ rapidly increases to 67.3%. However, when the *r*_v_ continues to increase from 6 to 10, the improvement of *η*_v_ is not obvious and finally stabilizes at about 70%. The main reason for this trend is that when the velocity of the spray increases, the mean square velocity of droplet granules in the turbulent aggregation kernel increases. According to Equation (19), so the particle aggregation rate *a*(*d*_i_, *d*_j_) increases with the increase of the particle mean square velocity *U_i_*^2^, thereby improving the dust removal efficiency. On the other hand, the greater the mist spray speed, the shorter the time that droplets are in the air. This will negatively affect the capture of dust.

**Figure 10:**
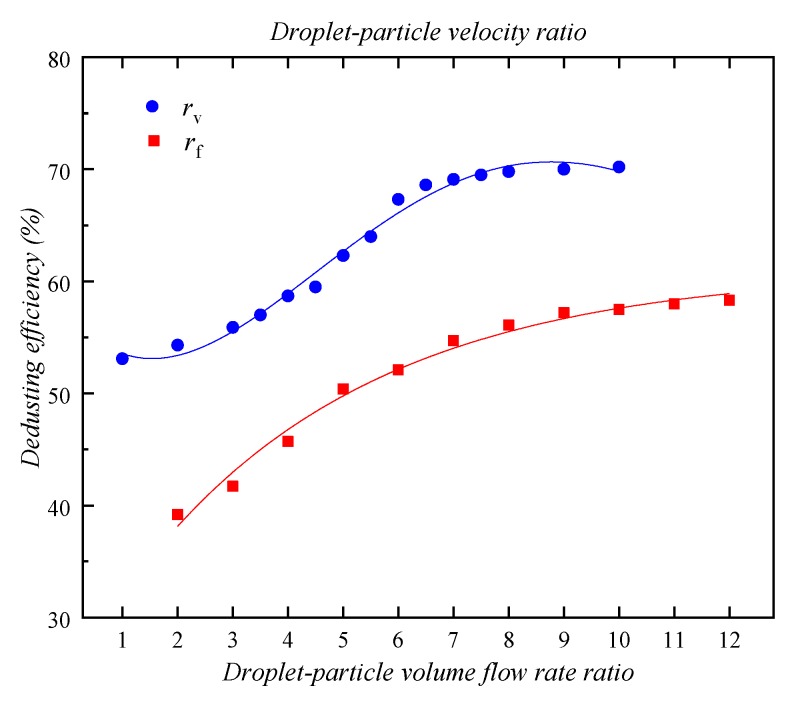
The fitted curves of *η* with respect to *r*_f_ and *r*_v_.

Similarly, the fitted formula for the dedusting efficiency *η*_v_ and the droplet–particle velocity ratio *r*_v_ can be obtained using Equation (36). Then Figure 10 shows the fitted curves of *η* with respect to *r*_f_ and *r*_v_.

(35) $$\eta_{f}=11.846\ln(r_{f})+30.375\;(R^{2}=0.9745)$$

(36) $$\eta_{v}=-0.0819r_{v}{}^{3}+1.2102r_{v}{}^{2}-2.4142r_{v}+54.451\;(R^{2}=0.986)$$

### 4.4. Effect of Ventilation Velocity

**Figure 11:**
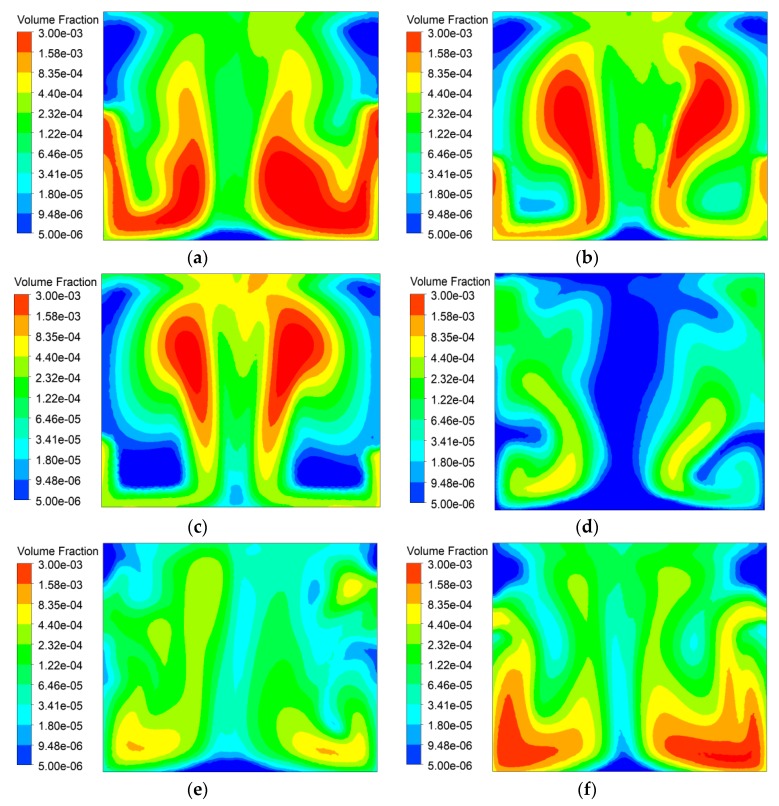
Distribution of dust particle volume fraction. (**a**) ventilation velocity of 1 m/s (**b**) ventilation velocity of 2 m/s. (**c**) ventilation velocity of 3 m/s (**d**) ventilation velocity of 4 m/s. (**e**) ventilation velocity of 5 m/s (**f**) ventilation velocity of 6 m/s.

**Figure 12:**
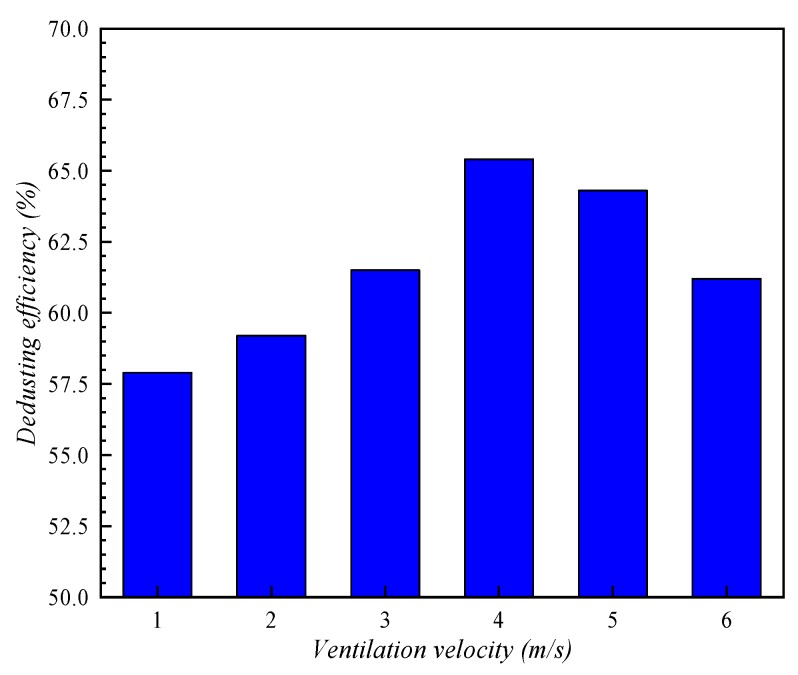
Comparison of dedusting efficiency at different ventilation velocities.

Figure 11 shows the distributions of the volume fraction of dust particles under the action of six groups of ventilation velocity on the plane *x* = 4.7 m, and the relationship between dedusting efficiency and ventilation velocity can be obtained, as shown in Figure 12. The figures show that when air inlet velocity increases from 1 m/s to 4 m/s, dedusting efficiency increases from 57.9% to 65.4%, but as the wind speed continues to increase, dedusting efficiency begins to decline. Moderate ventilation velocity can increase the probability of collisions between particles and the efficiency of turbulent aggregation. However, since the physical model is similar in shape to a roadway, when the flow velocity of airflow is too large, it will have a strong effect on the movement of dust and water mist particles, resulting in two kinds of particles with speeds in the same direction, thus reducing the relative speed between them and resulting in a negative impact on collision and aggregation.

## 5. Conclusions

In the work presented here, a mathematical model based on a two-fluid model for wet dedusting is proposed. The model considers in detail the aggregation between particles and droplets caused by turbulence, and also the deposition processes of dust particles caused by Brownian diffusion and the gravitational deposition. The PBM is applied to describe the spatiotemporal evolution of particle and droplet size distribution. Based on our analysis, the following conclusions can be drawn:

1. The proposed mathematical model is well validated by the detailed experimental results for spray dedusting in the existing literature, and the numerical results of dedusting efficiency agree well with the experimental values. The turbulent aggregation kernel and the deposition kernel proposed in this paper are accurate enough to describe the wet dedusting process dominated by respirable dust capture and deposition.
2. Key parameters analysis shows that the smaller the droplet diameter of the water mist formed by the atomizing device, the higher the capture efficiency of respirable dust. In other words, in practical application the dedusting efficiency of the respirable dust can be increased by ensuring that the water mist diameter is between 15 μm and 70 μm. The volume–flow rate ratio *r*_f_ and velocity ratio of droplets to dust *r*_v_ also have great influence on dedusting efficiency.
3. When *r*_f_ increases from 2 to 12, the dedusting efficiency increases from 39.2% to 54.7%. However, when this value continues to increase, the dedusting efficiency is not significantly improved. The fitting formula is given as *η_f_* = 11.846ln(*r_f_*) + 30.375, *R*^2^ = 0.9745.
4. When *r*_v_ increases 10 times, the dedusting efficiency increases from 53.1% to 70.2%. Increasing the ratio clearly has little effect on dedusting efficiency. The fitting formula is *η_v_* = −0.0819*r_v_*^3^ + 1.2102*r_v_*^2^ − 2.4142*r_v_* + 54.451, *R*^2^ = 0.9745.
5. An optimized air distribution should be considered in the dedusting efficiency. Under the ventilation condition with 4 m/s inlet air, spray dedusting reaches the highest efficiency.

The model proposed in this study fully accounts for the interactions between particles and the influence of air distribution, which is relevant for guiding and optimizing the selection and arrangement of dedusting equipment in practical engineering applications and therefore has broad application prospects. On the basis of these results, future study and research will investigate how to combine water-spray dedusting and ventilation dedusting more effectively so that they can be more widely used in practical projects.

## Figures and Tables

**Table 1:** Summary of proposed aggregation kernels in literatures.

Kernel	References	Expression	Comments
Free molecular aggregation	Smoluchowski (1917) [35]	$a_{f}=\frac{2k_{B}T}{3\mu }\frac{{\left(d_{i}+d_{j}\right)}^{2}}{d_{i}d_{j}}$	Very small particles (up to 1 μm) aggregate because of collisions due to Brownian motions; the frequency of collision is size-dependent
Coulomb aggregation	Williams and Loyalka (1991) [36]	$a_{c}=\frac{z(e^{z}+1)}{2(e^{z}-1)}a_{f}$ $z=\frac{q_{1}q_{2}}{2\pi k_{B}T\varepsilon_{0}\left(d_{i}+d_{j}\right)}$	The precondition is that the particle itself is already charged, no matter it is the same charge or different charge
Turbulence aggregation	Saffman and Turner (1956) [37] Zaichik and Alipchenkov (2001,2008) [38,39]	For the viscous subrange: $a_{t}=\varsigma_{T}\sqrt{\frac{8\pi }{15}}\gamma \frac{{\left(d_{i}+d_{j}\right)}^{3}}{8}$, for the inertial subrange: $a_{t}=\varsigma_{T}2^{3/2}\sqrt{\pi }\frac{{\left(d_{i}+d_{j}\right)}^{2}}{4}\sqrt{\left(U_{i}^{2}+U_{j}^{2}\right)}$	In the turbulent flow field, aggregation can occur by two mechanisms: viscous subrange mechanism and inertial subrange mechanism
Bubble aggregation	Wang et al. (2019) [40]	$a_{b}=\left\{ \begin{array}{ll} 0.5658\pi V_{b}{\left(\frac{d_{i}}{2}+\frac{d_{j}}{2}\right)}^{2}\sin^{2}\theta_{w} & for\;V_{ij}\leq V_{\max}\\ 0 & otherwise \end{array}\right.$	Aggregation will occur during a collision of two bubbles provided that the contact time exceeds the aggregation time required for drainage of the liquid film between them to a critical rupture thickness
Droplet aggregation	Williams et al. (2019) [41]	$a_{d}=\varepsilon \frac{\dot{\gamma }}{\pi }{\left(v_{i}^{1/3}+v_{j}^{1/3}\right)}^{3}$	Droplet centers are assumed to move along streamlines, and aggregation occurs when the distance between the droplets is less than the sum of their radii

**Table 2:** Data of droplet granularity from nozzles of different types in the literature.

Nozzles	D10 (μm)	D50 (μm)	D90 (μm)	Q (L/min)	Dedusting Efficiency (%)
1	34.93	70.62	117.67	6.46	53.1
2	41.10	70.62	109.22	7.92	32.3
3	38.57	74.67	122.81	7.36	61.2
4	32.94	65.15	112.39	8.15	45.5
5	37.01	69.03	118.36	5.63	48.6
6	40.03	75.14	123.85	4.30	39.7

**Table 3:** Of dedusting efficiency at different droplet-particle volume flow rate ratios.

Droplet-Particle Volume Flow Rate Ratio	Dedusting Efficiency (%)	Droplet-Particle Volume Flow Rate Ratio	Dedusting Efficiency (%)
2	39.2	8	56.1
3	41.7	9	57.2
4	45.7	10	57.5
5	50.4	11	58.0
6	52.1	12	58.3
7	54.7	—	—

**Table 4:** Of dedusting efficiency of different droplet–particle velocity ratio.

Droplet-Particle Velocity Ratio	Dedusting Efficiency (%)	Droplet-Particle Velocity Ratio	Dedusting Efficiency (%)
1	53.1	6	67.3
2	54.3	6.5	68.6
3	55.9	7	69.1
3.5	57.0	7.5	69.5
4	58.7	8	69.8
4.5	59.5	9	70.0
5	62.3	10	70.2
5.5	64.0	—	—
